# Clear Cell Squamous Cell Carcinoma of the Maxillary Gingiva Associated with *PIK3CA* and *HRAS* Mutations: Report of a Case and Literature Review

**DOI:** 10.1007/s12105-023-01580-8

**Published:** 2023-09-21

**Authors:** Katsutoshi Hirose, Takumi Shibahara, Akari Teramoto, Yu Usami, Sawako Ono, Yuri Iwamoto, Shumei Murakami, Kaori Oya, Narikazu Uzawa, Daisuke Motooka, Yumiko Hori, Eiichi Morii, Satoru Toyosawa

**Affiliations:** 1 Department of Oral and Maxillofacial Pathology, Osaka University Graduate School of Dentistry, 1-8 Yamadaoka, Suita, Osaka 565-0871 Japan; 2 Department of Oral & Maxillofacial Oncology and Surgery, Osaka University Graduate School of Dentistry, 1-8 Yamadaoka, Suita, Osaka 565-0871 Japan; 3 Department of Pathology and Medicine, Graduate School of Medicine, Dentistry and Pharmaceutical Sciences, Okayama University, Okayama, 700-8558 Japan; 4 Department of Oral and Maxillofacial Radiology, Osaka University Graduate School of Dentistry, 1-8 Yamadaoka, Suita, Osaka 565-0871 Japan; 5 Clinical Laboratory, Osaka University Dental Hospital, 1-8 Yamadaoka, Suita, Osaka 565-0871 Japan; 6 Genome Information Research Center, Research Institute for Microbial Diseases, Osaka University, 3-1 Yamadaoka, Suita, Osaka 565-0871 Japan; 7 Department of Pathology, Osaka University Graduate School of Medicine, 2-2 Yamadaoka, Suita, Osaka 565-0871 Japan; 8 Department of Central Laboratory and Surgical Pathology, National Hospital Organization, Osaka National Hospital, 2-1-14 Hoenzaka, Chuo-ku, Osaka 540-0006 Japan

**Keywords:** Squamous cell carcinoma, Clear cell squamous cell carcinoma, Oral tumor, *PIK3CA*, *HRAS*

## Abstract

**Background:**

Squamous cell carcinoma (SCC) is the most common oral malignancy, and somatic mutations in some driver genes have been implicated in SCC development. Clear cell SCC (CCSCC) is a rare histological variant of SCC, and various clear cell neoplasms must be considered in the differential diagnosis of CCSCC in the oral cavity. Based on a limited number of CCSCC cases reported in the oral cavity, CCSCC is considered an aggressive variant of SCC with a poor prognosis; however, its genetic characteristics remain unknown.

**Methods:**

A maxillary gingival tumor in an 89-year-old female was described and investigated using immunohistochemical staining, special staining, fluorescence in situ hybridization, and next-generation sequencing (NGS) with a custom panel of driver genes, including those associated with SCC and clear cell neoplasm development.

**Results:**

Histopathological examination revealed a proliferation of atypical epithelial cells with abundant clear cytoplasm and enlarged and centrally placed round nuclei. The tumor was exophytic with deep, penetrating proliferation. The atypical clear cells were continuous with the conventional SCC cells. Immunohistochemical analysis showed that the clear cells were positive for CK AE1/AE3 and CK5/6 and nuclear-positive for p63. In contrast, the clear cells were negative for αSMA, S100, HMB45, Melan-A, CD10, and p16. p53 immunoreactivity exhibited a wild-type expression pattern. Additionally, the clear cells were positive for periodic acid-Schiff (PAS) and negative for diastase-PAS, mucicarmine, and Alcian blue. Based on these results, the diagnosis of CCSCC was confirmed. Molecular analysis of the clear cells identified *PIK3CA* p.E542K (c.1624G>A) and *HRAS* p.G12A (c.35 G>C) somatic mutations classified as oncogenic. No pathogenic variants were identified in *TP53*, *EWSR1*, *AKT1*, *PTEN*, *BRAF*, *KRAS*, *NRAS*, *RASA1, or MAML2*.

**Conclusions:**

We report a case of CCSCC of the oral cavity with *PIK3CA* and *HRAS* mutations. The identification of *PIK3CA* and/or *HRAS* mutations is rare in SCC; however, both mutations are important potential targets for antitumor therapy. A detailed analysis of gene mutations in CCSCC may lead to a better understanding of its biological behavior and an improved prognosis, as well as a differential diagnosis from other clear cell neoplasms.

## Introduction

Squamous cell carcinoma (SCC) is the most common malignancy of the oral cavity [1]. Oral SCC (OSCC) is SCC that arises from the oral mucosal epithelium and has different histological subtypes, including basaloid, verrucous, spindle cell, papillary, adenosquamous, acantholytic, and caniculatum variants [1]. Clear cell SCC (CCSCC) is a rare histological variant of SCC and is characterized by the presence of abundant clear cytoplasm [2]. Kuo first described CCSCC of the skin [3], and Frazier et al. reported CCSCC of the oral cavity [4]. Clear cell change occurs extremely rarely in mucosal SCC, and only 12 cases of CCSCC developed in the oral cavity have been reported to date, including the present case [4–16] (Table 1). Based on a limited number of CCSCC cases reported in the oral cavity, CCSCC has been purported to be an aggressive variant of SCC that has a poor prognosis [9, 11–13] (Table 1).

**Table 1:** Clinical characteristics of clear cell squamous cell carcinoma cases in the oral cavity origin

Case	Author	Age/sex	Location	Recurrent	Metastasis	Follow-up
1	Frazier et al. [4]	59/F	Mandibular gingiva	N/A	N/A	Lost
2	Kumar et al. [7]	70/F	Anterior maxilla and right mandibular (2 sites)	N/A	LN	Died within 2 months
3	Nainani et al. [8]	52/M	Buccal mucosa	N/A	LN	Died within 3 months
4	Kaliamoorthy et al. [9]	35/F	Lateral tongue and lingual vestibule	N/A	No	N/A
5	Khoury et al. [10]	66/F	Tongue to the floor of the mouth	N/A	Lung (3 months later)	N/A
6	Devi et al. [11]	55/M	Maxillary alveolar ridge	N/A	LN	Alive 5 months
7	Katoti et al. [12]	59/M	Upper jaw	N/A	N/A	N/A
8	Ramani et al. [13]	42/F	Mandibular alveolar mucosa	+ (6 months later)	N/A	Lost
9	Hasegawa et al. [14]	70/M	Tongue	+	LN and Lung (3 months later)	N/A
10	Mukkanwar et al. [15]	60/M	Posterolateral border of the tongue	N/A	N/A	Lost
11	Mahamad Apandi et al. [16]	65/M	Floor of the mouth	+ (26 months later)	Lung (34 months later), LN (38 months later)	N/A
12	Present case	89/F	Maxillary alveolar ridge	+ (3 months later)	LN (8 months later)	Died within 8 months

*F* female, *M* male, + positive, − negative, *N/A* data not available, *LN* lymph node

Activation of phosphatidylinositol 3-kinase (PI3K)/AKT and RAS/RAF signaling pathways, which regulate cell proliferation and growth, apoptosis, autophagy, invasion, and migration, is observed in various malignancies, including SCC [17, 18]. Mutations in the genes involved in signaling pathways are closely related to cancer development and prognosis [17, 18]. Currently, efforts are focused on understanding the molecular and cellular consequences of these mutations and the opportunities for targeted therapies [18–21]. Detailed analysis of genetic mutations in pathways involving potential targets for antitumor therapy may lead to an improved prognosis for CCSCC. However, no reports identifying genetic mutations in CCSCC exist, and the genetic characteristics are still unknown. Moreover, various clear cell neoplasms may be found in the oral cavity, which must be considered in the differential diagnosis of CCSCC [6]. These clear cell neoplasms that are independent of SCC have well-defined genetic profiles that may help specify diagnoses in difficult cases (Table 2). Here, we report a case of CCSCC of the maxillary gingiva with *PIK3CA* and *HRAS* mutations and review the literature on CCSCC of the oral cavity.

**Table 2:** Staining panel and molecular findings for the differential diagnosis of clear cell neoplasms in the oral cavity

	CK	p63	SMA	S100	Melan-A	CD10	d-PAS	Molecular findings
Clear MEC	+	+	−	−	−	−	+	*CRTC1/3::MAML2*
Clear MEca	+	+	+	+	−	−	−	*EWSR1* rearrangement *PLGA1* rearrangement
HCCC	+	+	−	−	−	−	−	*EWSR1::ATF1* *EWSR1::CREM*
CCOC	+	+	−	−	−	−	−	*EWSR1::ATF1, EWSR1::CREM*
Malignant melanoma	−	−	+	+	+	−	−	*BRAF* mutation
CCRCC	+	−	−	−	−	+	−	
Present case	+	+	−	−	−	−	−	*PIK3CA* mutation *HRAS* mutation

*CK*, cytokeratin; *SMA*, smooth muscle actin; *d-PAS*, diastase-periodic acid-Schiff; *MEC*, mucoepidermoid carcinoma; *CRTC1/3*, CREB regulated transcription coactivator 1/3; *MAML2*, mastermind-like transcriptional coactivator 2; *MEca*, myoepithelial carcinoma; *EWSR1*: Ewing sarcoma breakpoint region 1; *PLGA1*, pleomorphic adenoma gene 1; *HCCC*, hyalinizing clear cell carcinoma; *ATF1*, activating transcription factor 1; *CREM*, cAMP responsive element modulator; *CCOC*, clear cell odontogenic carcinoma; *BRAF*, B-Raf proto-oncogene, serine/threonine kinase; *CCRCC*, clear cell renal cell carcinoma; *PIK3CA*, phosphatidylinositol-4, 5-bisphosphate 3-kinase catalytic subunit alpha; *HRAS*, HRAS proto-oncogene, GTPase

## Case Report

### Clinical Summary

**Fig. 1:**
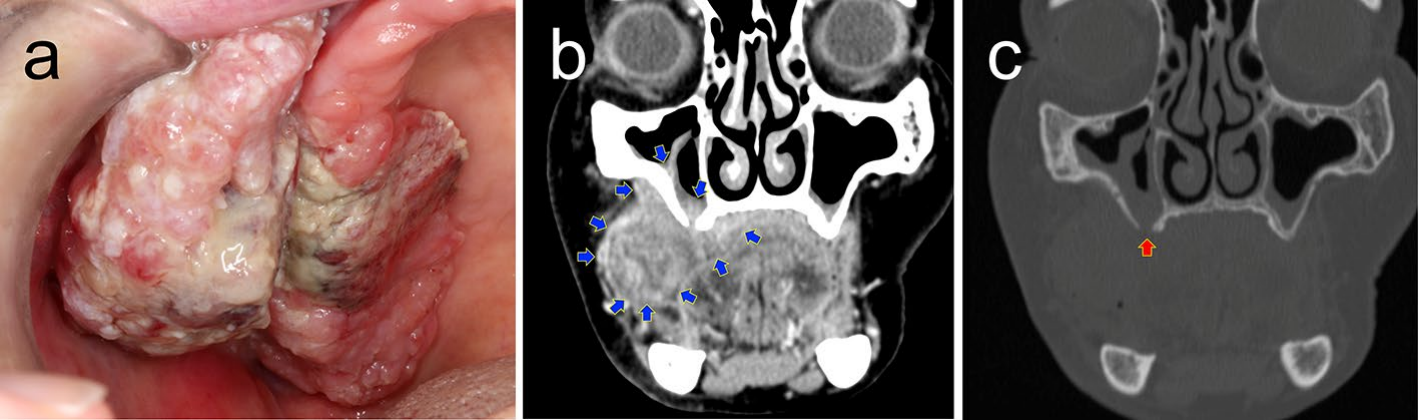
Clinical presentation. **a** Intraoral finding. Representative coronal computed tomography (CT) images with bone window (**b**) and with contrast-enhanced (**c**). The blue arrows indicate a large tumor extension (**b**). The red arrow indicates a bone penetration at the alveolar process of the right maxilla (**c**)

**Fig. 2:**
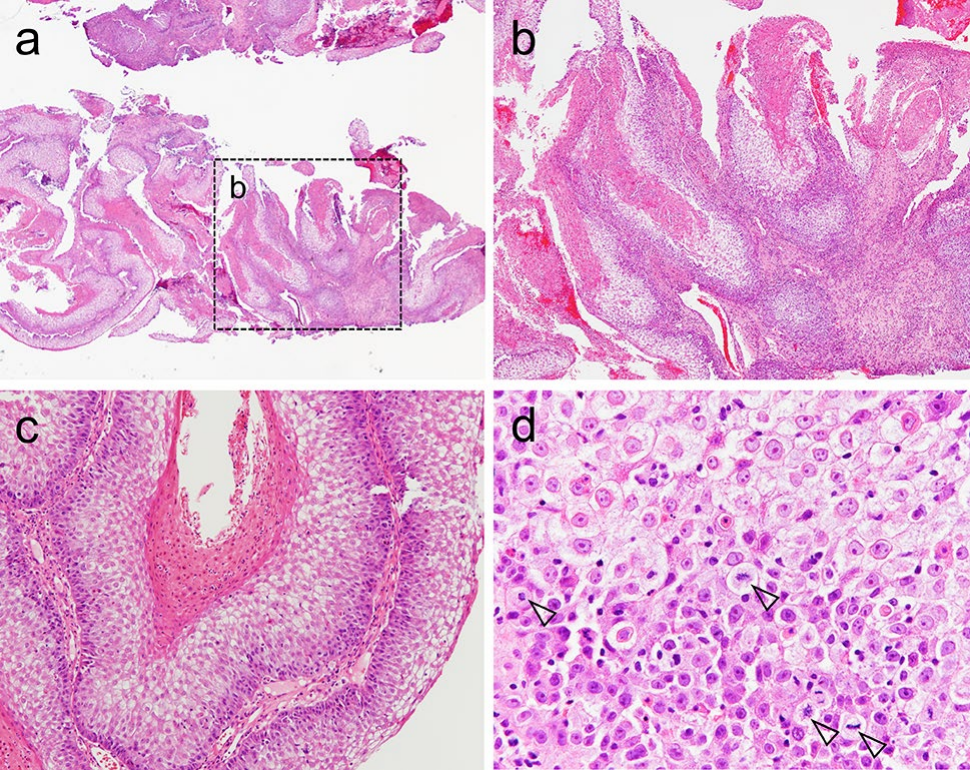
Histological findings of the biopsy. The exophytic tumor mass showed a penetrating growth pattern, resulting in several deep crypts filled with keratin debris (**b** was the black dotted-box area in **a**). **c** The tumor cells featured abundant clear cytoplasm, especially from the parabasal cells to the surface epithelium. **d** Clear cells exhibited enlarged and centrally placed round nuclei and nuclear and cellular atypia. Arrowheads indicate mitotic figures

**Fig. 3:**
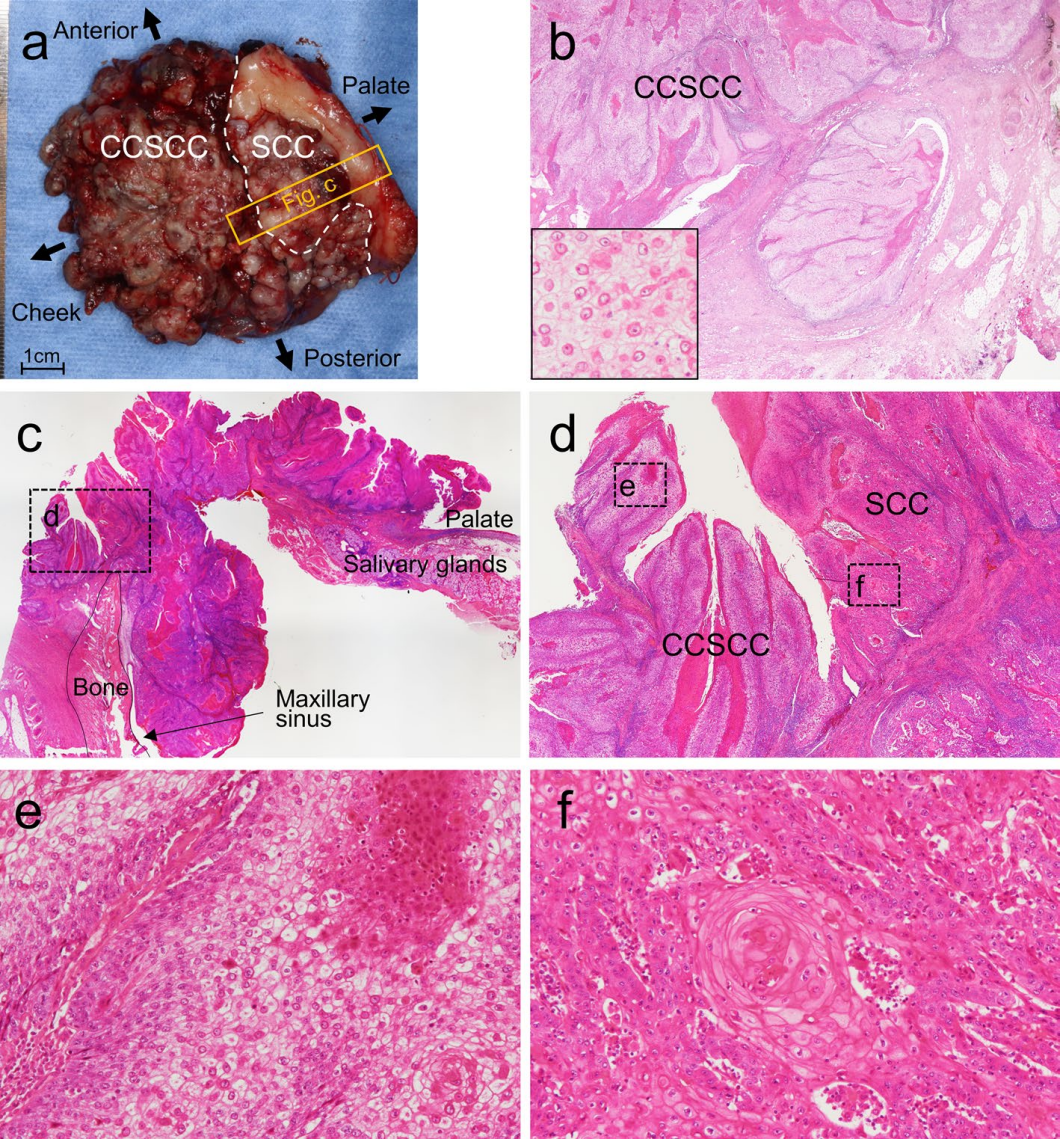
Histological findings of the surgical specimen. **a** Gross view of the surgical specimen. **b** Region of clear cell squamous cell carcinoma (CCSCC). CCSCC infiltrated deep connective tissue (inset: cytology of CCSCC). **c**, **d** Transitional area between CCSCC (left side) and conventional squamous cell carcinoma (SCC) (right side). **c** shows the orange lined-box area in **a**. **d** shows the black dotted-box area in **c**. **e, f** Region of CCSCC or SCC in the transitional area (both from the black dotted-box area in **d**)

An 89-year-old female was referred to the Osaka University Dental Hospital for painful swelling in the upper right gingival region. The patient had noticed a gingival mass for 1 month and had no significant medical history. Intraoral examination revealed an approximately 60 × 40 mm lobulated mass with an ulcerative surface on the right maxillary posterior gingiva extending to the buccal mucosa (Fig. 1a). Computed tomography (CT) revealed an infiltrative lesion with maxillary bone resorption (Fig. 1b, c). With a provisional diagnosis of SCC, an incisional biopsy was performed (Fig. 2) followed by segmental maxillectomy (Fig. 3). The tumor recurred after 3 months, and the patient died 8 months after surgery due to complications related to disease recurrence (Table 1).

### Pathological Findings

Biopsy showed an infiltrative neoplasm (Fig. 2a). The tumor was primarily exophytic but with a pattern of deep, pushing invasion with keratin debris in crypt spaces (Fig. 2a, b). Tumor cells had prominent clear cytoplasm, especially from the parabasal cells to the surface epithelium (Fig. 2c). Clear cells exhibited enlarged, centrally placed, round nuclei (Fig. 2d). Nuclear and cellular atypia of tumor cells were observed (Fig. 2d). Based on these observations, a clear-cell variant of SCC was suspected, and subsequent segmental maxillectomy was performed (Fig. 3a).

**Fig. 4:**
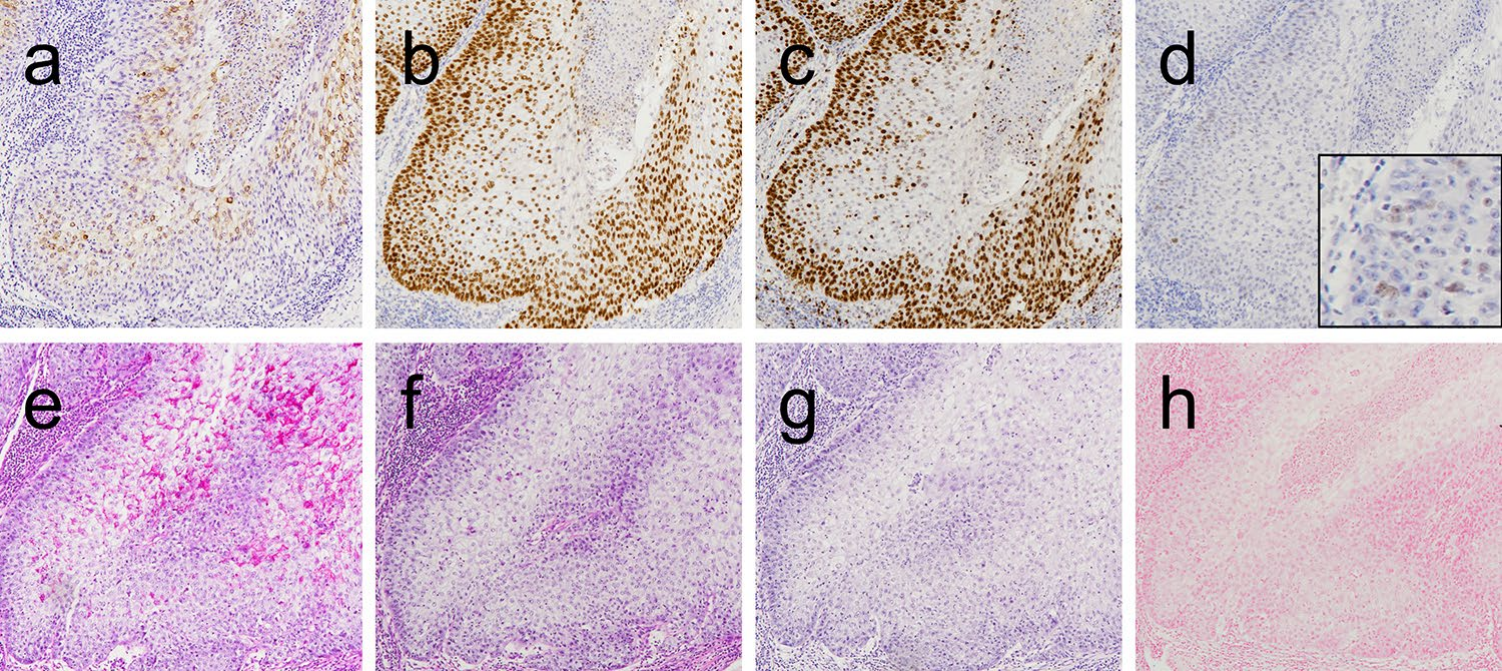
Immunohistochemical and special stains. **a** CK AE1/AE3, **b** p63, **c** Ki-67, and **d** p53 immunostains. **e** Periodic acid-Schiff (PAS), **f** PAS with diastase, **g** mucicarmine, and **h** Alcian blue stains

Histopathological examination of the surgical specimen affirmed biopsy findings. Additionally, sheets and islands of atypical clear cells infiltrated deep connective tissues (Fig. 3b). The clear cells were contiguous with conventional SCC cells connected to the normal epithelium (Fig. 3c–f), indicating the clear cells to be a component of the SCC. Clear cells accounted for a majority (80%) of tumor cells (Fig. 3a). Conventional SCC extended from the oral surface to the maxillary sinus with bone resorption (Fig. 3c). We did not perform immunohistochemical staining, special staining, or molecular analysis of the conventional SCC since the conventional SCC was observed only in formalin-fixed paraffin-embedded (FFPE) samples containing bone and decalcified by formic acid (Fig. 3c). Immunohistochemical analysis showed that the clear cells were positive for both CK AE1/AE3 (Fig. 4a) and CK5/6 mainly in the upper half of the epithelial layers, and were nuclear-positive for p63 in all epithelial layers (Fig. 4b). In contrast, the clear cells were negative for αSMA, S100, HMB45, Melan-A, CD10, and p16. The clear cells exhibited a p53 wild-type expression pattern (negative to weakly positive) (Fig. 4d). Immunohistochemical evaluation of Ki-67 revealed nuclear staining, mostly in the basal and parabasal cells of the atypical epithelium (Fig. 4c). Additionally, the cytoplasm of the clear cells was positive for periodic acid-Schiff (PAS) and negative for diastase-PAS, mucicarmine, and Alcian blue, suggesting accumulation of glycogen in the cytoplasm of the atypical clear cells (Fig. 4e–h).

Postoperative positron emission tomography (PET) CT was performed to assess distant metastasis. No signs of tumors were detected in other organs. Therefore, the final diagnosis of CCSCC of the maxillary gingiva was established.

### Molecular Analysis

**Fig. 5:**
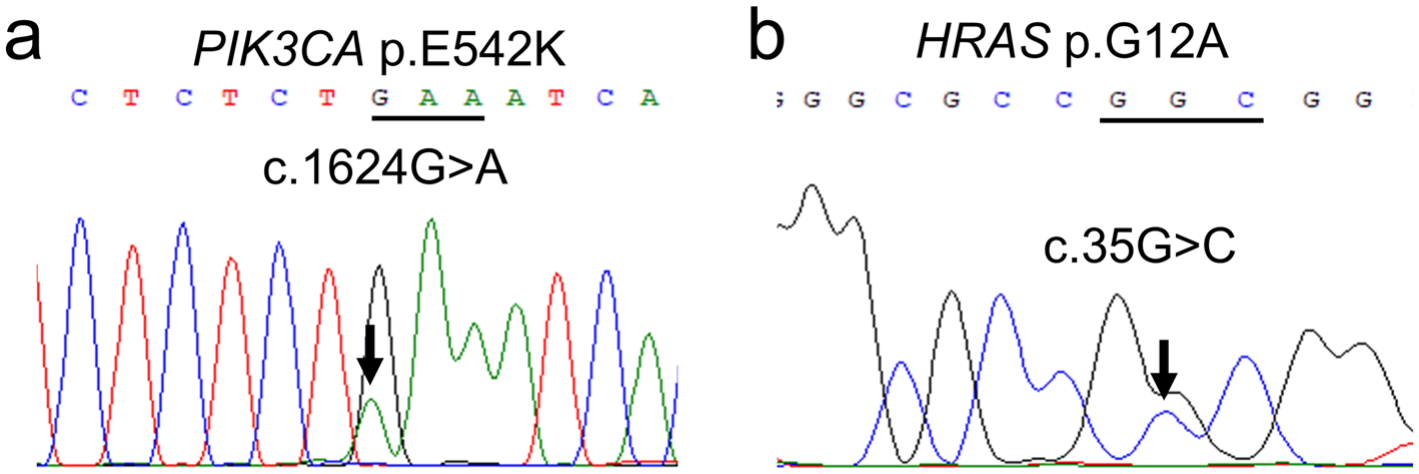
Molecular analysis. Direct gene sequencing shows chromatograms for *PIK3CA* p.E542K (c.1624G>A) (**a**) and *HRAS* p.G12A (c.35G>C) (**b**)

To further investigate the genetic profile of CCSCC, targeted next-generation sequencing (NGS) was performed using a custom panel as previously described [22]. The gene panel was designed using SureDesign (https://earray.chem.agilent.com/suredesign) to cover the whole *EWSR1* gene (coverage 90.91%), and entire exons of *TP53* gene or genes associated with the PI3K/AKT and RAS/RAF signaling pathways (*PIK3CA*, *AKT1*, *PTEN*, *BRAF*, *KRAS*, *NRAS*, *HRAS*, and *RASA1*). FFPE samples, in which tumor cells comprised approximately 60% of the total cells, were selected, and DNA was obtained from the sample. Polymerase chain reaction (PCR) assays and direct sequencing were performed to confirm gene mutations. Sequencing identified *PIK3CA* p.E542K (c.1624G>A) and *HRAS* p.G12A (c.35G>C) somatic mutations classified as oncogenic (Fig. 5a, b). No pathogenic variants were identified in *TP53*, *EWSR1*, *AKT1*, *PTEN*, *BRAF*, *KRAS*, *NRAS*, or *RASA1*. Moreover, *MAML2* rearrangement was not detected by fluorescence in situ hybridization (Z-2014-50, ZyoVision, Bremerhaven, Germany) (data not shown).

## Discussion

CCSCC of the oral cavity is rare, and its genetics relative to conventional SCC are unclear. To date, only 12 cases of CCSCC in the oral cavity have been reported, including our case [4, 7–16]. Previous reports have described that, histologically, CCSCC cells show abundant clear cytoplasm along with enlarged and centrally placed round nuclei [7, 9–11, 14]. It has been suggested that the proportion of clear cells required to define CCSCC is > 25% [2, 5]. Our case was consistent with the definition of CCSCC, both in the cytopathological findings and the proportion of clear cells in the lesion. Thus, our case is the first case of CCSCC, to our knowledge, in which a gene mutation has been described.

In the oral cavity, it is necessary to distinguish CCSCC from other tumors composed of clear cells: salivary gland carcinomas (clear cell variant mucoepidermoid carcinoma, clear cell myoepithelial carcinoma, and hyalinizing clear cell carcinoma [HCCC]), odontogenic carcinoma (clear cell odontogenic carcinoma [CCOC]), malignant melanoma, and metastatic carcinoma [6, 23] (Table 2). The lack of intracellular mucin, confirmed by d-PAS, mucicarmine, and Alcian blue staining results, excluded the diagnosis of mucoepidermoid carcinoma [6]. The lack of myoepithelial markers (such as SMA and S100) excluded clear-cell myoepithelial carcinoma [6]. The tumor location on the oral surface and the histology of squamous differentiation excluded HCCC and CCOC [6, 23]. The lack of S100, Melan-A, and HMB45 immunoreactivity excluded malignant melanoma [6, 23]. Metastatic tumors, such as clear cell renal cell carcinoma (CCRCC), were excluded because there were no signs of tumors in the other organs and CD10 immunoreactivity was absent [6]. These pathological and clinical findings led to the diagnosis of CCSCC in our case. Besides, our case of CCSCC was genetically different from other clear cell neoplasms in that *MAML2* rearrangement (characteristic of mucoepidermoid carcinoma), *EWSR1* rearrangement/translocation (characteristics of clear cell variants of myoepithelial carcinoma, HCCC, and CCOC), and *BRAF* mutations (detected in malignant melanoma and odontogenic tumors) were not detected (Table 2) [6, 23]. On the other hand, both *PIK3CA* (p.E542K) and *HRAS* (p.G12A) oncogenic mutations were detected, which are hardly detected in SCC (Fig. 5a, b) [1]. Thus, genetically, CCSCC may be an entity of SCC and distinct from other clear cell neoplasms in the oral cavity.

*PIK3CA* and *RAS* (*KRAS*, *NRAS*, and *HRAS*) mutations activate the PI3K/AKT and RAS/RAF pathways, respectively [17, 18]. Both pathways are critical drivers of tumorigenesis and potential targets for antitumor therapy [17–21]. Oncogenic mutations in *PIK3CA* and *RAS* have been identified in various malignancies, and both occasionally coexist [24]. However, in head and neck SCC (HNSCC), including OSCC, most genetic mutations are associated with tumor suppressor genes such as *TP53*, and genetic mutations in the PI3K/AKT or RAS/RAF pathways are rare [1, 25–32]. Kobayashi et al. reported that the most frequently mutated gene among 284 HNSCC cases was *TP53* (67%), followed by *PIK3CA* (8%), *AKT1* (4%), and *HRAS* (3%) [27]. Among HNSCC cases, only one had both *PIK3CA* and *HRAS* mutations [27]. In OSCC, the mutation frequency of *PIK3CA* ranges from 0 to 13.92% [30–32]. No significant correlation was found between *PIK3CA* mutations and survival rates in HNSCC and OSCC [27, 30–32]. Mutation frequencies of *HRAS* in OSCC range from 5 to 17.4% [30–32]. Carrying an *HRAS* mutation is considered a high-risk factor for poor prognosis and survival in HNSCC and OSCC [31–33]. HNSCC with *HRAS* mutations shows poor clinical outcomes with a high recurrence rate following primary definitive treatment (50–67% recurrence within 6 months), short disease-free survival (4.0 months; 95% CI 1.0 to 36.0), and overall survival (15.0 months; 95% CI 6.0 to 52.0) [33]. In this context, CCSCC recurred 3 months after primary resection in our patient, who had clear surgical margins at the time of resection, and the patient subsequently died 8 months later from complications related to tumor recurrence. Further studies are required to determine the association between *HRAS* mutations and poor prognoses in other CCSCC cases.

Several PI3K/AKT and RAS/RAF targeting agents are currently undergoing clinical trials, and molecular profiling of these targets needs to be investigated [17–21]. A recent study demonstrated that tipifarnib, a farnesyltransferase inhibitor that disrupts HRAS function, dramatically improved clinical outcomes in patients with *HRAS*-mutant HNSCC [21]. Moreover, PI3K inhibitors have demonstrated antiproliferative, pro-apoptotic, and antitumor activities in a range of preclinical cancer models as a single agent or in combination with other anticancer therapies [19, 20]. CCSCC of the oral cavity is considered an SCC variant [8, 11, 13–15]. Thus, the PI3K/AKT and RAS/RAF pathways may be important potential targets for future therapeutic options in patients with CCSCC.

## Conclusion

In conclusion, we report a case of CCSCC in the oral cavity associated with *PIK3CA* and *HRAS* mutations, potential targets for antitumor therapy. A detailed analysis of gene mutations in CCSCC may lead to a better understanding of its biological behavior and an improved prognosis, as well as distinguish CCSCC from other clear cell neoplasms.

## Data Availability

The surgical materials and datasets analyzed in the current study are available from the corresponding author upon reasonable request.

## Abbreviations

AKT1: AKT serine/threonine kinase 1
BRAF: B-Raf proto-oncogene, serine/threonine kinase
HCCC: Hyalinizing clear cell carcinoma
CCOC: Clear cell odontogenic carcinoma
CCRCC: Clear cell renal cell carcinoma
CCSCC: Clear cell squamous cell carcinoma
CT: Computed tomography
EWSR1: Ewing sarcoma breakpoint region 1
HNSCC: Head and neck squamous cell carcinoma
HRAS: HRAS proto-oncogene, GTPase
KRAS: KRAS proto-oncogene, GTPase
MAML2: Mastermind-like transcriptional coactivator 2
NGS: Next-generation sequencing
NRAS: NRAS proto-oncogene, GTPase
OSCC: Oral squamous cell carcinoma
PAS: Periodic acid-Schiff
PCR: Polymerase chain reaction
PET-CT: Positron emission tomography-computed tomography
PI3K: Phosphatidylinositol 3-kinase
PIK3CA: Phosphatidylinositol-4, 5-bisphosphate 3-kinase catalytic subunit alpha
PTEN: Phosphatase and tensin homolog
RASA1: RAS P21 protein activator 1
SCC: Squamous cell carcinoma
